# Usefulness of the Core Outcome Measures Index in Daily Clinical Practice for Assessing Patients with Degenerative Lumbar Disease

**DOI:** 10.1155/2012/474685

**Published:** 2012-03-05

**Authors:** Carlos Lozano-Álvarez, Daniel Pérez-Prieto, Guillem Saló, Antoni Molina, Andreu Lladó, Manuel Ramírez

**Affiliations:** Department of Orthopaedic Surgery and Traumatology, Parc de Salut Mar, Hospitales del Mar y la Esperanza, 08003 Barcelona, Spain

## Abstract

*Introduction*. Outcome evaluation is an important aspect of the treatment of patients with degenerative lumbar disease. We evaluated the usefulness of the Core Outcome Measures Index (COMI) in assessing people affected by degenerative lumbar disease in daily clinical practice. *Methods*. We evaluated 221 patients who had completed preoperatively and 2 years after surgery VAS pain, Short Form-36 (SF-36), Oswestry Disability Index (ODI) and COMI. We calculated the change of scores and its sensitivity to change. The internal consistency of the COMI items and the correlation between the COMI scores and the scores of the other measurements were assessed. *Results*. Statistically significant differences were observed between the mean scores of the preoperative and 2 years questionnaires for nearly all measurements. COMI showed a good internal consistency, except for the preoperative pain subscale. The sensitivity to change was high for the total COMI and its pain and well-being subscales and moderate for the rest. The COMI demonstrated strong correlation with the other measurements. *Conclusions*. The COMI is a useful tool for assessing the patient-based outcome in the studied population. Given its simplicity, good correlation with the SF-36 and ODI and its good sensitivity to change, it could replace more cumbersome instruments in daily clinical practice.

## 1. Introduction

Degenerative lumbar disease (DLD) and chronic low back pain (CLBP) are orthopaedic problems of the highest incidence in the Spanish population [1]. In the United States, the lifetime prevalence of low back pain has been reported to be as high as 84% and the prevalence of CLBP to be about 23%, with 11-12% of the population being disabled by low back pain [2]. Often, DLD and CLBP require surgical intervention so that DLD has become the leading cause of arthrodesis in the spine [1]. In the USA, the annual number of lumbar fusions for degenerative lumbar disease has increased from 174,223 in 1998 to 413,171 in 2008 [3].

Patient-based outcomes may be the most important tool clinicians, patients, and policymakers can use to identify the effectiveness of different low back pain treatments. In 1998, a multinational group of back pain investigators designed the Core Outcome Measures Index (COMI) to evaluate pain, function, generic health status or well-being, disability, and satisfaction [4]. The COMI ultimate goal was to provide a standardized outcome assessment without an excessive burden of instruments or questions that make it difficult for patients to complete the instruments of evaluation. The COMI was validated against well-validated instruments such as the Roland-Morris or the Oswestry Disability Index (ODI) for back specific function and the Medical Outcomes Study Short Form-36 (SF-36), its Short Form SF-12, or the EuroQol for general health status. In 2006, a Spanish group validated the Spanish version of the COMI [5]. The authors designed a prospective study that aimed to evaluate the reliability, validity, and responsiveness of this instrument. They evaluated this instrument in patients with subacute osteoporotic fracture (quick improvement of the pain after treatment) and chronic low back pain (slow improvement of the pain) and related the COMI scores to the scores of the Spanish-validated ODI, SF-36, and SF-12. They concluded that the COMI was a useful tool to evaluate patient-based outcomes when the respondent burden is an important problem. Still, subscale scores needed to be further tested in other populations.

 The objective of our study is to evaluate the usefulness of the COMI as an outcome measurement in daily clinical practice for patient suffering from DLD.

## 2. Material and Methods

### 2.1. Patient Sample

We reviewed the outcomes from 263 patients operated between 2005 and 2008 for degenerative lumbar disease. Of those 263 patients who had completed the preoperative questionnaires, 221 also completed the questionnaires 2 years after surgery. Thirty-five of the 42 patients without postoperative outcomes could not be found, and 7 had died.

Patients were excluded if they were younger than 18 years old, had surgeries for infectious disease, tumours, or rheumatic origin, or had a language barrier that prevented them from properly understanding the questionnaires. We included all patients older than 18 years old who were operated for the following diagnoses: degenerative disc disease, stenosis, disc herniation, spondylolisthesis, and pseudarthrosis. Epidemiological data collected during the study were age, sex, employment status, diagnosis, surgical procedure, and degree of comorbidity on the American Society of Anaesthesiologists (ASA) scale [6, 7].

### 2.2. Questionnaires

 All patients were clinically evaluated and then self-completed the validated Spanish version of the SF-36 version 2 [8, 9] to evaluate their general health, the validated Spanish version of the ODI [10, 11] to assess their disability, visual analogue scales (VAS) [12, 13] to evaluate lumbar and sciatic pain, and the validated Spanish version of the COMI [4, 5] used to comprehensively evaluate patients. All questionnaires were filled out before surgery and 2 years after surgery.

 The COMI [4, 14] is a questionnaire composed of 8 questions that evaluates pain (2 items), function (1 item), well-being (1 item), disability (2 items) and satisfaction (2 items). The scores of the questionnaire range from 1 to 5, with 1 being the best possible result. The total COMI score is the average of the 5 dimensions. It was designed for a simpler but effective standardized evaluation of outcome in patients with low back pain and would replace more cumbersome health-related questionnaires in daily practice.

### 2.3. Data Analysis

Statistical analyses were performed with SPSS 15.0 (SPSS Inc., Chicago, IL, USA). Student's *t*-tests were used to compare the pre-operative and post-operative scores; a *P* value below 0.05 was considered statistically significant. The change in scores from pre-operative to 2-year follow-up was calculated as the preoperative scores minus the post-operative scores. A negative change score indicates improvement for ODI, COMI, and VAS, while a positive change score indicates improvement for the SF-36. The magnitude of change (sensitivity to change) was assessed by the standardized mean response (SMR). SMR is one of the possible calculations of effect size; specifically it is obtained by dividing the mean difference by the standard deviation of the change scores [15]. The use of effect size allows for comparisons between different outcome measures because it translates score differences into a standard unit of measurement. Applying Cohen's threshold values of effect size to SMR, sensitivity is considered trivial for SMR values lower than 0.20, small for SMR values between 0.20 and 0.50, moderate for SMR values between 0.50 and 0.80, and large for SMR values greater than 0.80 [16].

 The internal consistency of the various questionnaires was evaluated with Cronbach's alpha test. Cronbach's alpha was not applicable for the function and well-being subscales of the COMI because they are composed of a single item. Alpha values between 0.8 and 0.9 indicate a good consistency, and values between 0.7 and 0.8 indicate an acceptable level of consistency [17].

Construct validity was assessed through Pearson's correlation. Items measuring similar concepts were expected to have high correlation coefficients (>0.6), and items measuring different concepts were expected to have low correlation coefficients (<0.4) [5].

## 3. Results

The mean patient age was 55.1 years (22 to 86 years), and 112 patients (50.7%) were women. At the time of surgery, 53.8% of the patients were employed, 24.9% were on disability, 18.1% were retired, and 3.2% were unemployed (Table 1).

The most frequent causes of surgical intervention were degenerative disc disease (DDD, 38.9% of cases) and lumbar spinal stenosis (34.3%). The most common surgical treatments were transforaminal lumbar interbody fusion (TLIF, 36.2%) and posterolateral fusion (32.1%). The average degree of comorbidity measured by ASA scale was 2 with a range from 1 to 4 (Table 1).

### 3.1. Magnitude of Change and Responsiveness

There was an overall improvement in the average scores of the different questionnaires from the preoperative visit to the visit at 2 years, and this difference was statistically significant in all measures except for the mental health subscale of the SF-36 (Table 2). The SMR indicated a high sensitivity to change for the total COMI and its pain and well-being subscales. The sensitivity to change was moderate for the COMI function, disability, and satisfaction subscales (Table 2).

### 3.2. Internal Consistency

Cronbach's alpha indicated a good internal consistency of the COMI both in the preoperative phase (alpha = 0.807) and the 2-year evaluation (alpha = 0.91), except for the preoperative pain subscale (Table 3). The low internal consistency of the pain subscale may be due to the fact that it is a combination of back pain and leg pain while the patients in our sample were not likely to evenly suffer from back and leg pain.

### 3.3. Construct Validity

Pearson's correlation coefficients indicated that the COMI total score and its subscales had a statistically significant correlation with almost all values of the ODI, SF-36, and VAS before surgery and after surgery and the score difference (Table 4). In general, items measuring similar concepts had a high (>0.6) correlation coefficient, for instance, the total COMI and ODI (*r* = 0.7) or the disability scale of the COMI and ODI (*r* = 0.6). Items measuring different concepts had low (<0.4) correlation coefficients, for example, the COMI satisfaction scale and the PCS of the SF-36 (*r* = −0.2) or the COMI well-being scale and the PCS (*r* = −0.2). However, this trend was not consistent for all measures of similar/dissimilar concepts (e.g., *r* = 0.5 between COMI pain scale and lumbar VAS) and for measurements times (e.g., *r* = 0.5 between preoperative COMI pain scale and lumbar VAS while *r* = 0.8 between postoperative COMI pain scale and lumbar VAS).

## 4. Discussion

 In this study, the COMI demonstrated good internal consistency, validity, and responsiveness to change in our patient population. Its brevity makes it easier for patients to answer. Its simple scoring and free availability simplifies its administration. For all these reasons, the COMI appears a useful measurement tool of patients' outcomes in daily practice.

The COMI was originally designed by a multinational group as a standardized core of questions that assess briefly but globally patients based outcomes [5]. The design took into account factors such as breadth of coverage, demonstrated validity and reproducibility, and demonstrated responsiveness, practicality (brevity and low cost), compatibility with widely promoted instruments or batteries, and importance to patients and society. The resulting COMI is comprised of 5 scales already validated and in use in some form in other instruments such as EuroQol, National Health Interview Survey, the North American Spine Society, and American Academy of Orthopedic Surgeons instruments.

 The psychometric characteristics of the COMI were established with a study of the COMI prospectively administered to 277 patients with low back pain. It demonstrated good reliability, reproducibility, validity, and sensitivity of the COMI composite score and subscales [18]. The German [14], French [19], and Italian [20] versions as well as a neck [21] version of the COMI have been validated. It is recommended as “*a suitable instrument for implementation in the Spine Tango Registry or in any other multi-language databases of outcomes in LBP patients*” … “*the systematic and widespread use of this version in similar settings might enhance the quality of the follow-up of patients with chronic LBP*” [20]. This instrument is considered in both versions as a practical, reliable, and valid tool and will be of value for international studies and surgical registries.

 In 2006, Spanish groups of the *Hospital Universitari Vall d'Hebron* (Barcelona) and *Fundación Jiménez Díaz* (Madrid) published the validation study of the Spanish COMI [5]. Their sample included two groups of patients (osteoporotic vertebral fracture and chronic low back pain), and outcomes were evaluated with the Spanish version of COMI and Spanish well-validated versions of SF-36, SF-12, and Oswestry Disability Index. The COMI showed good reproducibility, internal consistency, construct validity, and responsiveness, comparable to the more generally used outcome measurements.

 The present study examined the use of the COMI in patients with various spine pathologies as typically encountered in daily clinical practice. An important methodological limitation of our study is the lack of test-retest study to confirm the reproducibility of the COMI in our population. Our retrospective analysis did not allow for a test-retest study. Otherwise, just as in the Spanish, German, Italian, and French validation studies, the results of our studies showed similarly good internal consistency, construct validity, and sensitivity to change.

## 5. Conclusion

 The COMI is a valid and sensitive questionnaire for the evaluation of patients with degenerative lumbar disease before and after treatment. The results of this study confirm that the COMI is a short, time-saving, easily scored, and multidimensional instrument that can be widely used in daily clinical practice for assessment and monitoring of patients with degenerative lumbar disease.

## Figures and Tables

**Table 1:** Epidemiological data (221 patients).

Age years	(range)
55.1	(22–86)

Sex	*n* (%)
Female	112 (50,7)
Male	109 (49,3)

Employment situation	*n* (%)
Employed	119 (53,8)
Permanent disability	27 (12,2)
Temporary disability	28 (12.7)
Retired	40 (18,1)
Unemployed	7 (3,2)

ASA score	(range)
2	(1–4)

Diagnostics	*n* (%)
DDD	86 (38,9)
Lumbar stenosis	76 (34,3)
Disc herniation	28 (12,7)
Spondylolisthesis	16 (7,2)
Pseudarthrosis	15 (6,7)

Treatment	*n* (%)
TLIF	80 (36,2)
Posterolateral fusion	71 (32,1)
PLIF	31 (14,03)
Discectomy	28 (12,7)
Laminectomy	11 (4,97)

**Table 2:** Preoperative and 2-year postoperative scores and magnitude of change.

Health status measures	Preoperative		Postoperative		Difference		SMR
	Mean	SD	Mean	SD	Mean	SD	
ODI	45.60	17.85	36.60	22.24	−8.76*	19.43	−0.451
COMI	3,77	0.76	2.60	0.53	−1.07*	1.19	−0.899
Pain	3.88	0.89	2.79	1.27	−1.13*	1.28	−0.883
Function	3.99	1.12	2.90	1.53	−1.01*	1.68	−0.601
Well-being	4.83	0.47	3.19	1.49	−1.60*	1.48	−1.081
Disability	3.35	1.67	1.89	1.29	−1.01*	1.85	−0.546
Satisfaction	2.81	1.09	2.21	1.34	−0.61*	1.15	−0.530
SF36v2							
Physical Function (PF)	29.29	9.48	36.68	12.79	7.18*	12.05	0.596
Role physical (RP)	30.66	8.76	21.17	4.37	−9.79*	9.46	−1.035
Bodily pain (BP)	30.25	6.99	39.10	12.56	8.87*	12.54	0.707
General health (GH)	42.16	9.01	39.83	11.98	−1.90**	11.13	−0.171
Vitality (VT)	35.35	9.36	44.17	12.01	8.82*	11.69	0.755
Social function (SF)	30.45	13.71	39.68	13.89	8.56*	15.71	0.545
Role emotional (RE)	36.26	14.82	16.10	5.39	−20.38*	13.91	−1.465
Mental health (MH)	39.40	10.64	37.90	11.92	−1.09	1.77	−0.085
PCS	30.90	7.41	36.66	10.89	6.38*	10.9	0.585
MCS	39.91	12.28	32.78	9.76	−6.10*	12.50	−0.488
VAS							
Back	7.55	2.15	5.40	3.41	2.01*	3.34	0.601
Sciatica	7.62	2.62	4.18	3.47	2.39*	3.81	−0.627

*P < 0.001, **P < 0.05.

Sensitivity to change (SMR): low *≈* 0.2; moderate *≈* 0.5. High > 0.8.

**Table 3:** Internal Consistency (cronbach's alpha coefficient).

	*α*	
	Preoperative	Postoperative
ODI	0.854	0.915
COMI (Total)	0.807	0.910
Pain	0.446	0.776
Function	—	—
Well-being	—	—
Disability	0.911	0.749
Satisfaction	0.827	0.858
SF-36v2		
PF	0.888	0.938
GH	0.750	0.839
RP	0.889	0.938
RE	0.879	0.932
SF	0.662	0.856
BP	0.768	0.887
VT	0.828	0.811
MH	0.862	0.763

*α* > 0.7-0.8 (max value = 1) → good reliability.

**Table 4:** Pearson's correlation coefficients.

		ODI	SF-36v2										VAS	
			PF	RP	BP	GH	VT	SF	RE	MH	PCS	MCS	Lumbar	Sciatica
Total	Before	**0.706**	**−0.549**	**−0.672**	**−0.728**	**−0.333**	**−0.568**	**−0.582**	**−0.397**	**−0.573**	**−0.610**	**−0.497**	**0.522**	**0.421**
	After	**0.340**	**−0.814**	**−0.714**	**−0.886**	**−0.678**	**−0.695**	**−0.759**	**−0.573**	**−0.577**	**−0.849**	**−0.538**	**0.823**	**0.642**
	Change	**0.728**	**−0.669**	**−0.486**	**−0.842**	**−0.466**	**−0.491**	**−0.760**	−0.074	**−0.492**	**−0.724**	**−0.410**	**0.760**	**0.643**

Pain	Before	**0.460**	**−0.364**	**−0.375**	**−0.533**	**−0.230**	**−0.393**	**−0.238**	*−0.171*	**−0.336**	**−0.432**	**−0.234**	**0.521**	**0.675**
	After	**0.740**	**−0.726**	**−0.695**	**−0.821**	**−0.549**	**−0.600**	**−0.630**	**−0.468**	**−0.467**	**−0.789**	**−0.438**	**0.755**	**0.740**
	Change	**0.483**	**−0.573**	**−0.481**	**−0.644**	*−0.246*	**−0.377**	**−0.482**	−0.112	**−0.319**	**−0.552**	−0.193	**0.758**	**0.751**

Fuction	Before	**0.583**	**−0.448**	**−0.537**	**−0.644**	**−0.275**	**−0.453**	**−0.429**	**−0.216**	**−0.377**	**−0.580**	**−0.301**	**0.476**	**0.425**
	After	**0.750**	**−0.739**	**−0.638**	**−0.788**	**−0.682**	**−0.629**	**−0.702**	**−0.560**	**−0.502**	**−0.753**	**−0.491**	**0.647**	**0.538**
	Change	**0.575**	**−0.470**	**−0.499**	**−0.693**	**−0.405**	**−0.422**	**−0.461**	−0.124	*−0.208*	**−0.563**	−0.159	**0.655**	**0.624**

Well-being	Before	**0.235**	*−0.167*	**−0.215**	**−0.223**	**−0.212**	**−0.174**	*−0.145*	−0.126	**−0.233**	**−0.210**	*−0.163*	*0.205*	0.094
	After	**0.675**	**−0.667**	**−0.566**	**−0.729**	**−0.644**	**−0.598**	**−0.573**	**−0.490**	**−0.497**	**−0.707**	**−0.465**	**0.615**	**0.407**
	Change	**0.464**	**−0.470**	**−0.230**	**−0.605**	**−0.462**	**−0.370**	**−0.215**	−0.109	*−0.190*	**−0.568**	−0.039	**0.396**	*0.374*

Disability	Before	**0.627**	**−0.443**	**−0.607**	**−0.529**	**−0.237**	**−0.470**	**−0.618**	**−0.342**	**−0.493**	**−0.472**	**−0.471**	**0.369**	0.219
	After	**0.707**	**−0.613**	**−0.567**	**−0.710**	**−0.517**	**−0.536**	**−0.631**	**−0.527**	**−0.364**	**−0.682**	**−0.399**	**0.627**	**0.556**
	Change	**0.531**	**−0.452**	**−0.593**	**−0.585**	*−0.359*	*−0.348*	**−0.515**	−0.093	**−0.364**	**−0.522**	−0.277	0.458	*0.560*

Satisfaction	Before	**0.228**	*−0.174*	**−0.252**	**−0.300**	**−0.217**	**−0.269**	**−0.260**	*−0.163*	**−0.276**	**−0.229**	**−0.246**	*0.256*	*0.274*
	After	**0.566**	**−0.549**	**−0.509**	**−0.620**	**−0.522**	**−0.469**	**−0.610**	**−0.450**	**−0.435**	**−0.553**	**−0.405**	**0.570**	**0.402**
	Change	**0.326**	**−0.452**	−0.205	**−0.413**	−0.161	−0.175	**−0.502**	−0.088	−0.203	**−0.522**	−0.277	**0.535**	*0.367*

Statistical significance (*P*):  *P* < 0.05 (italics). *P* < 0.01 (bold).
